# Attributes, Methods, and Frameworks Used to Evaluate Wearables and Their Companion mHealth Apps: Scoping Review

**DOI:** 10.2196/52179

**Published:** 2024-04-05

**Authors:** Preetha Moorthy, Lina Weinert, Christina Schüttler, Laura Svensson, Brita Sedlmayr, Julia Müller, Till Nagel

**Affiliations:** 1 Department of Biomedical Informatics Center for Preventive Medicine and Digital Health Medical Faculty Mannheim, Heidelberg University Mannheim Germany; 2 Institute of Medical Informatics Heidelberg University Hospital Heidelberg Germany; 3 Section for Oral Health, Heidelberg Institute of Global Health Heidelberg University Hospital Heidelberg Germany; 4 Medical Center for Information and Communication Technology University Hospital Erlangen Erlangen Germany; 5 Institute for Medical Informatics and Biometry, Carl Gustav Carus Faculty of Medicine Technische Universität Dresden Dresden Germany; 6 Human Data Interaction Lab Mannheim University of Applied Sciences Mannheim Germany

**Keywords:** wearables, mobile health, mHealth, mobile phone, usability methods, usability attributes, evaluation frameworks, health care

## Abstract

**Background:**

Wearable devices, mobile technologies, and their combination have been accepted into clinical use to better assess the physical fitness and quality of life of patients and as preventive measures. Usability is pivotal for overcoming constraints and gaining users’ acceptance of technology such as wearables and their companion mobile health (mHealth) apps. However, owing to limitations in design and evaluation, interactive wearables and mHealth apps have often been restricted from their full potential.

**Objective:**

This study aims to identify studies that have incorporated wearable devices and determine their frequency of use in conjunction with mHealth apps or their combination. Specifically, this study aims to understand the attributes and evaluation techniques used to evaluate usability in the health care domain for these technologies and their combinations.

**Methods:**

We conducted an extensive search across 4 electronic databases, spanning the last 30 years up to December 2021. Studies including the keywords “wearable devices,” “mobile apps,” “mHealth apps,” “physiological data,” “usability,” “user experience,” and “user evaluation” were considered for inclusion. A team of 5 reviewers screened the collected publications and charted the features based on the research questions. Subsequently, we categorized these characteristics following existing usability and wearable taxonomies. We applied a methodological framework for scoping reviews and the PRISMA-ScR (Preferred Reporting Items for Systematic Reviews and Meta-Analyses extension for Scoping Reviews) checklist.

**Results:**

A total of 382 reports were identified from the search strategy, and 68 articles were included. Most of the studies (57/68, 84%) involved the simultaneous use of wearables and connected mobile apps. Wrist-worn commercial consumer devices such as wristbands were the most prevalent, accounting for 66% (45/68) of the wearables identified in our review. Approximately half of the data from the medical domain (32/68, 47%) focused on studies involving participants with chronic illnesses or disorders. Overall, 29 usability attributes were identified, and 5 attributes were frequently used for evaluation: satisfaction (34/68, 50%), ease of use (27/68, 40%), user experience (16/68, 24%), perceived usefulness (18/68, 26%), and effectiveness (15/68, 22%). Only 10% (7/68) of the studies used a user- or human-centered design paradigm for usability evaluation.

**Conclusions:**

Our scoping review identified the types and categories of wearable devices and mHealth apps, their frequency of use in studies, and their implementation in the medical context. In addition, we examined the usability evaluation of these technologies: methods, attributes, and frameworks. Within the array of available wearables and mHealth apps, health care providers encounter the challenge of selecting devices and companion apps that are effective, user-friendly, and compatible with user interactions. The current gap in usability and user experience in health care research limits our understanding of the strengths and limitations of wearable technologies and their companion apps. Additional research is necessary to overcome these limitations.

## Introduction

### Background

Wearable technology, also known as *wearable devices*, includes smart electronic devices worn in close proximity to the surface of the human body. These devices can detect, analyze, and transmit information concerning body signals such as vital signs and physiological data, including step count and heartbeat [1-3]. Smart wearable technologies and their high-performance microsensors are of growing importance for patient health monitoring and are being widely accepted into clinical use and trials [4-7]. These technologies have the capability to amplify personal wellness and raise awareness in the spectrum of preventive health care. Consumers continue to rely on smart devices such as mobile phones and smartwatches to engage in healthy behavior [8-10]. They also assist in the self-management of chronic conditions, preventive measures, and aftercare, for example, diabetes monitoring [11], rehabilitation [12-14], fall detection [15,16], wound healing [17], and even monitoring symptoms of long-term illness [18-20]. Wearable technology further enhances the continuum of care within interdisciplinary communication and improves individuals’ health and well-being, all in their natural mobile environment [3,21].

### Commercial Wearables Versus Medical Wearables

The growing demand for health care technology, particularly wearable devices, has led to the proliferation of various medical and smart health care wearables. However, there is ambiguity in distinguishing between commercial consumer wearable devices and wearable medical devices. The European Union regulations [22] define medical devices as those intended for medical purposes such as disease diagnosis, monitoring, treatment, injury management, and physiological process modification; however, this scope does not include wearable technologies, such as smartwatches, smart bands, and mobile phone–based devices, designed primarily to provide users as tools for health monitoring and management. Fotiadis et al [23] defined wearable medical devices as self-contained, noninvasive devices with specific medical functions. Although a clear definition of wearable medical wearables remains elusive, these devices serve as a convergence point for both conventional medical device manufacturers and consumer-oriented companies aiming to enter the profitable medical market. The traditional distinction between medical and consumer-grade devices relies on the primary intention; however, we found that many commercial devices are being used opportunistically in health care and clinical trials.

Despite the expanding scope of wearable devices, unresolved concerns persist among general consumers regarding the safety, security, and usability of these devices [24-28]. Therefore, ensuring the fit-for-use of these technologies for specific users in clinical settings must be ascertained. The assessment of the usability of these technologies is critical to the success and adoption of wearable and mobile technology or the combination thereof. The identification and consideration of the appropriate attributes and methods for the measurement of usability as early on in the product development process can increase productivity, reduce errors, reduce user training and user support, and improve efficacy, thereby further broadening the acceptance of wearable and mobile technology by users [29-31].

### Definition of Usability

In the literature, the definition of *usability* varies, with some studies equating it to assess a device’s functionality, whereas others focus on aspects such as feasibility or performance. This ambiguity highlights the need for a comprehensive approach in measuring usability, considering users, devices, environment, and the actions users perform. The International Organization for Standardization (ISO) 9241 clarifies usability as “the extent to which a system, product or service can be used by specified users to achieve specified goals with effectiveness, efficiency, and satisfaction in a specified context of use” [32]. It is important to recognize that usability extends beyond the immediate outcomes of use. Established standards such as ISO 9241 or other regulatory frameworks primarily view usability as a result of use, emphasizing attributes such as effectiveness, efficiency, and satisfaction. However, a holistic evaluation of usability attributes that goes beyond immediate outcomes contributes to a deeper understanding of user interactions with these technologies and their acceptance in everyday use.

For better adoption of wearables in combination with their companion mobile health (mHealth) apps in clinical settings, usability needs to be considered to safeguard the effectiveness, functionality, and ease of use of these technologies. Concerning the acceptance of wearable technologies, it has been advocated that the devices must be easy to wear, affordable, possess suitable functions, and be appealing to users [33-35]. In such circumstances, designers, developers, and interdisciplinary researchers need to consider the development and use of such devices in a user-centered manner [36-38], thereby affirming that wearable technology is relative to the requirements of the users as it is a vital factor in the adoption of digital health apps and devices because it can be challenging for users owing to their health conditions. Furthermore, usability testing of these technologies allows researchers to understand how the wearable being developed meets users’ requirements before being used in health interventions.

### Exploring Key Attributes of Usability

Previous scientific literature disclosed the measurements of usability using different entities. These entities are defined as dimensions, components, scales, or factors of usability. According to Folmer and Bosch [39], these terms are analogous and hold the same meaning. Therefore, as defined by Wixon and Wilson [40], the term usability attribute is the characteristics of a product that can be measured. The most consistently reported usability attributes are effectiveness, efficiency, and satisfaction, which are part of the usability definition of ISO 9241-11:2018 [32,41]. Existing reviews have focused on reporting the usability attributes of mobile apps in health care; however, the shortcoming of applicable attributes for wearables and their companion app poses a challenge in assessing the usability of these technologies.

### Exploring Evaluation Methods of Usability

Usability assessment is instrumental in determining how well users learn and use technology to meet their goals. This includes the effectiveness and efficiency of a device and how satisfied the users are with the process. Therefore, different usability evaluation methods should be used to gather this information. Existing literature shows that different methods have been used for the testing of wearables such as field studies and laboratory experiments [42-44]. Although laboratory experiments, field studies, and hands-on measurement are some of the most commonly used methodologies, these are sometimes difficult to apply and have drawbacks. The prevailing usability methods assess different facets of usability, each providing different data. Accordingly, the selection of methods plays a pivotal role in evaluating the desired attributes of usability. Previous reviews have investigated the usability of wearables [45-47] and mHealth apps independently [41,48-50]. These reviews examined the usability assessment of wearables or mobile apps according to specific use cases in the health care domain [51-57]. Moreover, evaluation studies on the combination of the aforementioned devices were not taken into consideration. Research in this area continues to be fragmented, which demonstrates the importance of exploring further the requirements, functionalities, and capabilities of such wearable devices to enhance our comprehensive understanding of their use and acceptance.

### Objectives

This study aims to survey the existing literature in the field of medicine and health care that reports on the usability of wearable technology, mHealth apps, or their combination. Our scoping review seeks to analyze the literature in three ways: (1) type (commercial or medical) and category (stand-alone or paired) of wearable devices and their frequency of use in studies; (2) medical use cases; and (3) usability evaluation of these technologies, specifically usability attributes, methods, and frameworks.

## Methods

### Framework

This study uses the framework developed by Arksey and O’Malley [58] for reporting on scoping reviews, following the recommendations for enhancement of this approach by Levac et al [59]. We followed the five stages of the framework: (1) identifying the research question (RQ), (2) identifying relevant studies, (3) selecting studies, (4) charting the data, and (5) summarizing and reporting the results. In addition, the review followed the PRISMA-ScR (Preferred Reporting Items for Systematic Reviews and Meta-Analyses extension for Scoping Reviews) checklist to report the study selection process of the scoping review (Multimedia Appendix 1) [60,61].

### Stage 1: Identifying the RQs

For our scoping review, we used the PICO (Population, Intervention, Comparison, and Outcome) model [62,63] shown in Table 1 to help us regulate our RQs, outline the search strategy, and identify relevant studies within the health care domain. However, for our scoping study, the control or comparison aspect of the PICO methods was eliminated because our focus was not on comparative studies or controlled exposure.

**Table 1:** The PICO (Population, Intervention, Comparison, and Outcome) method applied to our review.

Aspect	Description	Our review
Patient, population, or problem	Problem to be addressed	Wearables Fitness trackers Physiological data
Intervention, prognostic factor, or exposure	Situation or condition or a characteristic of a patient (technology savvy). Exposure to be considered in treatments and tests	Mobile devices or smartphones Mobile apps
Control or comparison	Control or comparison intervention treatment or placebo or standard of care	Comparison is eliminated as the focus is not on comparative studies or controlled exposure
Outcome to measure or achieve	Outcome of interest—what can be accomplished, ensured, improved, or affected?	Usability and human factors

This scoping review aims to accomplish its objectives by answering the following RQs:

1. RQ1: What type (commercial or medical) and category (stand-alone or paired) of wearable devices and their companion mHealth apps were implemented and how frequently were they used in the studies?
2. RQ2: What medical use cases and medical data were reported?
3. RQ3: What usability methods, frameworks, and attributes were used for the usability evaluation?

### Stage 2: Identifying Relevant Studies

The focus of the second stage of the Arksey and O’Malley framework [58] was to find the relevant studies that match the RQs and the purpose of the scoping review. We began the review with an extensive search using keywords related to the PICO model. However, this raised questions about the sensitivity and specificity of the articles, that is, retrieving and identifying relevant research topic publications. Therefore, redefining the search terms after the initial search added the advantage of prioritizing the sensitivity of the relevant article.

We conducted our search in 4 electronic databases, including ACM Digital Library, IEEE Xplore, PubMed, and Web of Science (Clarivate Analytics), resulting in relevant studies covering the last 30 years up to December 2021. Relevant additional literature was also identified through other resources such as citations and expert recommendations. The search strategy was developed in association with the university librarian at the Medical Faculty Mannheim, Heidelberg University. The search integrated both search terms and Medical Subject Headings associated with the topics of health care, wearables, mHealth apps and terms used under the umbrella term *user experience*. The search strategy for the respective databases can be found in Multimedia Appendix 2.

### Stage 3: Study Selection

For our scoping review, all types of articles ranging from journal articles to conference papers were considered, without restrictions on the period of publication. In line with the systematic review methodology, we formulated inclusion and exclusion criteria for this scoping review. The inclusion and exclusion criteria are provided in Textbox 1. This allowed us to reduce the number of papers that were included in the screening of titles and abstracts. Citavi (version 6; Swiss Academic Software GmbH) was used for the collection of the articles.

**Inclusion and exclusion criteria for the scoping review.**

**Inclusion criteria**

- Language: English
- Papers focused on wearables or a prototype of wearables that have used usability testing for their evaluation, where methods such as questionnaires, observations, experimental testing, or surveys were used
- Papers focused on a mobile health (mHealth) app or a prototype of the mHealth app that used usability testing for its evaluation, where methods such as questionnaires, observations, experimental testing, or surveys were used
- Papers that use either a wearable, an mHealth app, or both in a medical use case, for example, chronic diseases
- Papers that use a user-centered design approach for developing wearables or mHealth apps

**Exclusion criteria**

- Inclusion criteria not fulfilled, for example, papers not written in English or not matching any of the secondary inclusion criteria
- Papers with the theme or topic of augmented reality and virtual reality, which may also include usability studies (eg, Google Glass)
- Papers that have only used audio and visual wearable aids (ie, without additional support from smartphones)
- Papers purely focused only on the technical aspects, technical descriptions, or features of wearables or mobile apps or PDAs in the development and testing processes of materials; self-developed sensors; or wearable sensors such as accelerometers, gyroscopes, or inertial measurement unit
- Papers that are focused mainly on medical professionals rather than patients, for example, describing algorithms or methods used for the optimization of viewing medical data (such as electrocardiogram and electroencephalogram)

We followed the recommendations from Daudt et al [64] for interdisciplinary teamwork in scoping reviews: we incorporated reviewers from different disciplines and backgrounds such as health services research, usability engineering, and medical informatics. The reviewers were divided into 2 groups such that each group had members with diverse backgrounds and expertise. Furthermore, an expert not involved in the screening reviewed mismatched publications from the groups and made discrete decisions for the inclusion and exclusion of articles. Each team member independently reviewed the titles, abstracts, and full text of the publications assigned to them. Studies were considered for the full-text reading if the inclusion criteria were met and cross-verified among team members.

### Stage 4: Charting the Data

At this stage, the data from the included studies were extracted. The review team collectively designed a structured data-charting format aligned with the RQs of the scoping review. Each team member individually extracted relevant characteristics from the included studies and adapted them to the data-charting format. Disagreements between the reviewers were resolved through discussions and feedback. The characteristics extracted from the included studies that are associated with the aim of this scoping review are as follows: (1) classification of wearable devices and mHealth apps (only wearables or paired); (2) type of wearable devices; (3) type of mHealth app (stand-alone or interactive); (4) medical use cases (if wearables or mobile apps or combination of both were used in a specific medical use case); (5) physiological data; (6) type of connections between wearables and apps (Wi-Fi or cables); (7) duration of usability studies; and (8) usability evaluation—usability attributes, frameworks, and methods. Excel (Microsoft Corp) was used to facilitate this process.

### Stage 5: Summarizing and Reporting the Results

To effectively summarize and organize the extracted data, a comprehensive search of the relevant literature was conducted to identify suitable articles to structure the examination and analysis. Two relevant literature sources were identified to fulfill our objectives. We aimed to find a suitable classification system that could effectively categorize various wearable devices. The classification proposed by Seneviratne et al [65], which provides a comprehensive survey of commercial wearable products grouped into 3 categories—accessories, e-textiles, and e-patches—served as a helpful tool for our analysis. In addition, we used the usability taxonomy hierarchy proposed by Alonso-Ríos et al [66] for our analysis. This taxonomy provided a comprehensive framework for organizing usability attributes in a logical and meaningful order.

We present our findings by integrating descriptive tables and graphical illustrations of the outcomes. These figures and graphics helped our analysis to directly connect the findings to the objectives of our review and identify the gaps in the literature. In our study, we illustrate the frequencies and percentages of the findings in coherent data visualizations, emphasizing the analysis and reporting of data and giving them a comprehensive meaning.

## Results

### Eligible Studies

**Figure 1:**
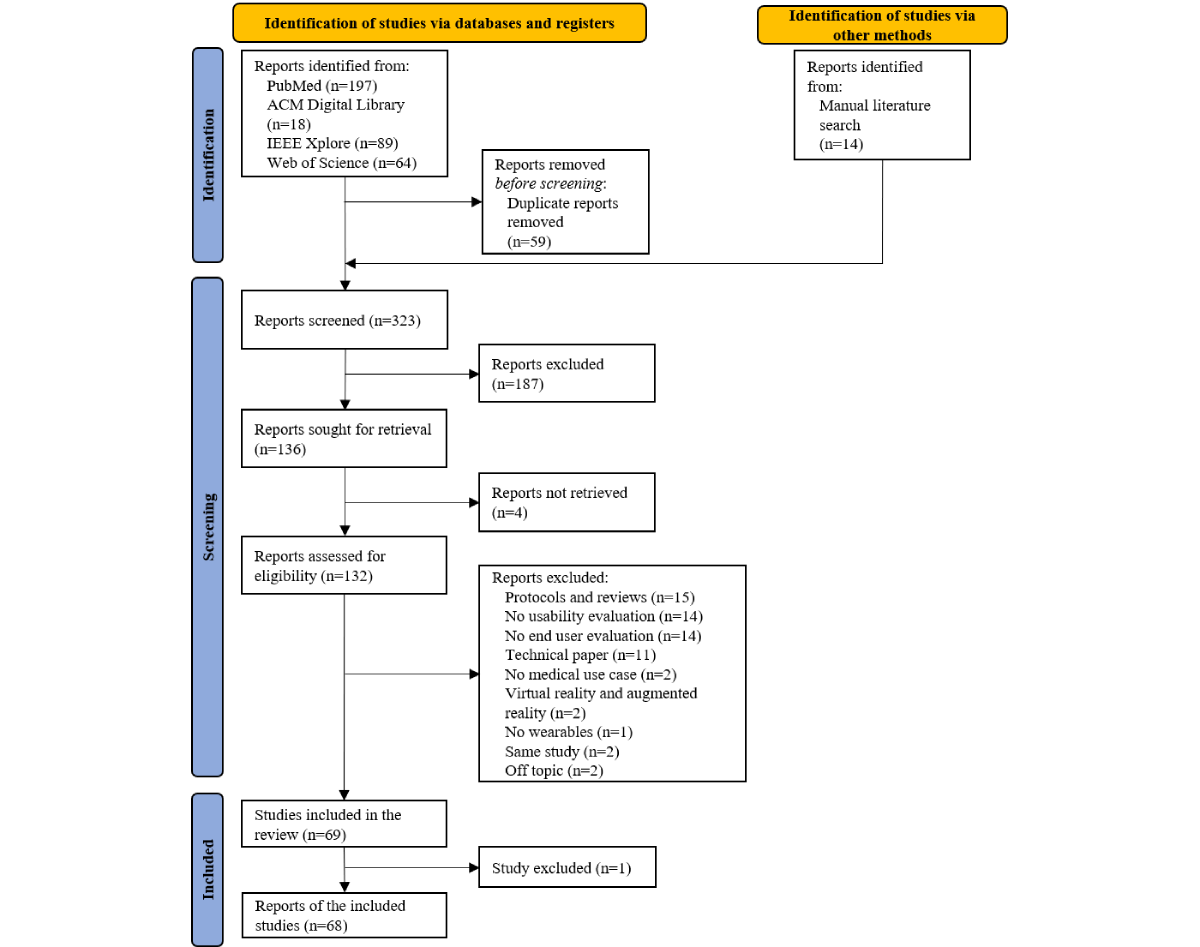
PRISMA-ScR (Preferred Reporting Items for Systematic Reviews and Meta-Analyses extension for Scoping Reviews) flow diagram of the selection process for the scoping review.

Our search yielded 382 records, including articles about wearables, mHealth apps, or their combination; research about the implementation of these technologies in medical use cases; and evaluations of their usability. Overall, 323 records were evaluated for the initial screening of titles and abstracts after eliminating duplicates. From these, 132 full-text papers were found, of which 62 were excluded, resulting in 69 studies whose data were charted per the study questions. Following the final text reading, a single study had to be excluded from this scoping review because it did not meet the predefined inclusion criteria, despite the presence of relevant keywords in the paper. The process of selection of articles for the scoping review can be seen in the PRISMA-ScR diagram shown in Figure 1.

### Wearable Devices and Their Frequency of Use

Most studies (57/68, 84%) used a combination of wearables and mobile apps. Overall, 12% (8/68) of the studies used wearables in conjunction with other technological devices such as smartphones, computers, recording devices, or PDAs; however, these devices were used independently from the wearables. Furthermore, the data extraction indicated that two-thirds of the studies (45/68, 66%) used commercially available wearables for their evaluation studies, of which approximately half of the studies (21/45, 47%) used Fitbit (Fitbit Inc.) devices as their source of data tracking and collection. Our data further showed that only 11% (5/45) of the studies used wearable devices that were certified as medical devices. In addition, only 18% (12/68) of the studies used a self-developed wearable prototype and a mobile app for data tracking and monitoring.

From the included studies, 9% (6/68) of the studies reported using only wearables in their studies. Of these 6 studies, 5 (83%) used commercially available wearable devices such as Fitbit and Samsung Gear S3 (Samsung Electronics Co, Ltd), whereas 1 (1%) study reported using a self-developed wearable prototype. However, 7% (5/68) of outliers were detected where 60% (3/5) of the studies mentioned using only a mobile app; 20% (1/5) of the studies reported using only a smartphone; and in 20% (1/5) of the studies, smartphone was used as a wearable device by attaching a 3D-printed phone holder around the user’s neck [67]. Although this is typically not defined as a wearable, the outcome of this study proved imperative in determining the different devices and variables used for evaluation. We used a classification system consisting of 6 distinct groups to categorize the diverse use of wearable technologies and mobile apps (with, without, or a combination thereof). They are (1) only wearables (eg, stand-alone wearables such as Fitbit and Garmin [Garmin Ltd]), (2) wearables+companion apps (eg, Garmin tracker+corresponding Garmin mobile app), (3) smartphone as wearables (eg, smartphone used in close proximity to the skin to track physiological data), (4) wearables+connectivity (not companion) apps (eg, wearables paired with connectivity apps for Bluetooth connection and not for presenting data), (5) wearables+other technologies (eg, Garmin+laptops, recording units, and PDAs), and (6) others (only smartphone or only app). The specified categories and the corresponding studies included in this review are presented in Table 2.

In most studies, the validation, accuracy, and certification of the used wearables were not thoroughly discussed, despite these aspects being considered essential in good research practices. Although some studies briefly touched upon validation or accuracy, they did not necessarily indicate that the wearables had undergone certification, such as Food and Drug Administration approval or Conformité Européenne mark. Authors of the included studies often omitted reporting the inaccuracies and validation limitations of consumer-grade wearables, particularly when usability was of significant importance. Instead, the accuracy of wearables was often assumed based on the authors’ validation of wearables’ selection through peer-reviewed research and their alignment with traditional instruments for measuring health data. The list of included studies along with the extracted information can be found in Multimedia Appendix 3 [43,67-133].

Most of the wearables (35/68, 51%) covered in the review were wrist worn, such as wristbands or smartwatches. However, only 9% (6/68) of the studies used multiple wearables, such as a smartwatch and chest belt or multiple wrist-worn wearables. Table 3 presents the data on the different categories of the types of wearable devices extracted from the included studies. More than half of the studies (48/68, 71%) deployed stand-alone mobile apps, which implies that users or patients collected health information using wearables and apps without sharing it with their professionals. Overall, 68% (46/68) of the studies determined that Bluetooth connections were the primary means of connectivity for wearables and the mobile apps that accompanied them. Data from the extracted studies revealed no linkage between the technologies; hence, outliers (4/68, 6%) were also recognized.

**Table 2:** Wearable devices, mobile apps, and their combination along with their frequency of use in studies (n=68).

Category of wearable devices and mobile apps	Frequency of use in studies, n (%)	References
Only wearables	6 (9)	[68-73]
Wearable devices+companion apps	49 (72)	[43,74-121]
Wearable devices+connected with apps (not companion app)	5 (7)	[122-126]
Wearables+other technologies (eg, laptops, recording units, and PDAs)	3 (4)	[127-129]
Smartphone as wearables	1 (1)	[67]
Others	4 (6)	[130] (smartphone only), [131-133] (app only)

**Table 3:** Categorization of the type of wearable devices according to the classification of wearable devices by Seneviratne et al [65] ordered by their frequency of use in studies (n=68).

Categorization of the type of wearables	Description	Devices	Studies, n (%)
Wrist worn	Wrist-worn devices with fitness tracking capabilities or other functionalities, generally without a touchscreen	Wrist bands	26 (38)
Wrist worn	Wrist-worn devices with a touchscreen display	Smartwatches	9 (13)
>1 wearable device in the study	Study includes >1 wearable device (any type)	Wrist bands, Upper arm bands, e-Patches, Sensor patches	6 (9)
Other accessories	Chest straps, belts, upper arm bands (in contrast to wrist-worn bands), or knee straps equipped with sensors for health tracking or other functionalities	Straps	5 (7)
e-Textiles	Main clothing items that also serve as wearables, such as shirts, pants, and undergarments	Smart garments	5 (7)
Hearables	Fits in or on an ear that contains a wireless link	Hearing devices	4 (6)
Other accessories	Clip-on	Clip-on	3 (4)
e-Patches	Sensor patches that can be adhered to the skin for either fitness tracking or haptic applications	Sensor patches	3 (4)
Outliers	Does not fit the categories	Wrist bands, Upper arm bands, e-Patches, Sensor patches	3 (4)
e-Textiles	Shoes, socks, insoles, or gloves embedded with sensors	Foot or hand-worn	2 (3)
Other accessories	Jewelry designed with features such as health monitoring and handless control	Smart jewelry	1 (1)
e-Patches	Tattoos with flexible and stretchable electronic circuits to realize sensing and wireless data transmission	e-tattoo or e-skin	—^a^
Head-mounted devices	Spectacles or contact lenses with sensing, wireless communication, or other capabilities	Smart eyewear	—
Head-mounted devices	Bluetooth enables headsets or earplugs. Sensor-embedded hats and neck-work devices are also found in research products	Headsets or earbuds	—

^a^Not available.

### Medical Use Cases and Reported Data

Our data showed that approximately half of the studies (32/68, 47%) focused on participants with chronic illnesses or disorders, indicating the importance of wearable technologies in managing and monitoring chronic conditions. The remaining 53% (36/68) of the studies encompassed various other medical use cases such as wellness, mental health, rehabilitation, sleeping disorders, otolaryngology, and preventive measures.

Most studies (40/68, 59%) routinely collected physiological data from users or patients. The most commonly collected health data revolved around physical activity, encompassing metrics such as steps taken, stairs climbed, and inertial measurement units. Approximately 35% (24/68) of the health data gathered in the studies focused on cardiac measurements, including electrocardiogram, heart rate variability, heart rate, or blood pressure. In addition to physical activity and cardiac measurements, other data types were also collected, albeit to a lesser extent. Sleep data accounted for 25% (17/68) of the collected information, and brain activity data, such as electroencephalogram recordings, constituted 4% (3/68) of the data. Furthermore, biosignals, including measurements such as skin conductance and respiration rate, were captured in 18% (12/68) of the studies. Other health data and observations such as acoustics, posture, blood glucose, and weight were also monitored in approximately 21% (14/68) of the cases, indicating the broad range of parameters that wearables can track and analyze.

In addition to physiological data, a small proportion of the studies (4/68, 6%) included in our analysis also collected nonphysiological data that were not directly linked to health parameters. These data contained various variables such as the number of cigarettes consumed per day, location data, and dietary intake. Although not directly related to traditional health measurements, the inclusion of such data provides a broader context and enables a more holistic understanding of individuals’ behaviors and lifestyle factors.

### Usability Attributes

The studies included in this review used various terms such as *usability characteristics* and *attributes*, which we consider to be synonymous. Therefore, we applied the usability attributes from the extracted data to the usability taxonomy [66]. We found that satisfaction (34/68, 50%), ease of use (27/68, 40%), user experience (16/68, 24%), perceived usefulness (18/68, 26%), and effectiveness (15/68, 22%) were the most commonly used attributes for assessing usability. Although user experience is acknowledged as the overarching term encompassing usability, conceptualizing user experience as a facet of usability captures the comprehensive perception arising from interactions with devices. This extends beyond the mere use of the device, encapsulating the entirety of the experience or the anticipated use of the technologies. Furthermore, we identified 32% (22/68) of the studies that simply reported usability or perceived usability. Moreover, our findings further indicate that out of the 29 identified usability attributes, 6 (21%) can be classified as quality attributes. Table 4 shows the mapping of the attributes identified in the review to the attributes defined in the usability taxonomy. These particular usability characteristics possess qualities that are directly related to the overall quality and performance of the technologies used in the studies. Table 5 presents the quality and product attribute matrix of the attributes ascertained in the review and the defined attributes from the ISO norm 25010 [134]. A detailed explanation of the different attributes in Tables 4 and 5 can be found in Multimedia Appendix 4 [66].

**Table 4:** Matrix mapping of the attributes identified in the scoping review and the usability taxonomy [66]^a^.

Usability attributes from scoping review	Usability taxonomy						
	Knowability	Operability	Efficiency	Robustness	Safety	Subjective satisfaction	Number of studies, n (%)
Accuracy		✓					5 (7)
Aesthetics						✓	7 (10)
Attitude						✓	4 (6)
Attractiveness						✓	2 (3)
Clarity	✓						1 (1)
Cognitive load			✓				2 (3)
Controllability		✓					1 (1)
Data quality		✓					1 (1)
Ease of use or perceived ease of use (effort expectancy, easiness, self-descriptiveness, and self-efficacy)	✓						27 (40)
Effectiveness (user errors, ease of executing a task, task completion, and task completeness)		✓					15 (22)
Efficiency (task time)			✓				6 (9)
Engagement						✓	7 (10)
Error tolerance				✓			1 (1)
Functionality		✓					7 (10)
Hedonic motivation						✓	2 (3)
Learnability	✓						4 (6)
Likes and dislike**s**						✓	1 (1)
Perceived usefulness (usefulness, utility, performance expectancy, and system usefulness)	✓						18 (26)
Satisfaction (subjective app quality, survey and ratings, positive and negative feedback, opinions and reactions, and participants’ experience)						✓	34 (50)
Technical difficulties				✓			2 (3)
Trust					✓		1 (1)
User control		✓					1 (1)
User experience (experience, overall subjective quality, and user-friendliness)						✓	16 (24)

^a^The rows list all the usability attributes identified in the scoping review and the columns list the first-level usability attributes from Alonso-Ríos et al [66], with each checkmark symbol indicating a match based on their description and their sublevel attributes. The last column lists the number of studies using the term from that row.

**Table 5:** Quality and product attributes identified in the scoping review that match the attributes from the International Organization for Standardization (ISO) norm ISO/International Electrotechnical Commission (IEC) 25010 [134]^a^.

Attributes identified in the scoping review	Quality attribute						Product attribute								
	Effectiveness	Efficiency	Satisfaction	Safety	Usability	Functional suitability		Performance efficiency	Compatibility	Usability	Reliability	Security	Maintainability	Portability	Number of studies (%)
Comfort			✓												8 (12)
Design—app interface and design and interface quality										✓					3 (4)
Effectiveness^b^	✓														15 (22)
Efficiency^b^		✓						✓							6 (9)
Facilitating conditions									✓						1 (1)
Information quality								✓							3 (4)
Interface quality										✓					2 (3)
Reliability											✓				1 (1)
Trust^b^				✓											1 (1)
Satisfaction^b^			✓												34 (50)

^a^The rows list all the attributes identified in the scoping review and the columns list the quality and product attributes from ISO norm 25010 [134], with each checkmark symbol indicating a match based on the description of the attributes. The last column lists the number of studies using the term from that row.

^b^Overlapping attributes also identified as usability attributes.

In addition, during the data extraction process, we obtained insights into the elements and factors that affect the evaluation of usability. Among the 68 included studies, 52 (76%) reported these elements and factors, which played a crucial role in shaping and guiding the measurement of usability. Notably, acceptance (21/68, 31%) emerged as the most commonly used element or factor in assessing usability, indicating its significance in understanding users’ acceptance and adoption of wearable technologies.

### Usability Evaluation Methods and Frameworks

Only 12% (9/68) of the studies outlined using some sort of framework for usability evaluation. User- or human-centered design (7/68, 10%) was the most commonly used framework. Our findings revealed that more than half of the studies (37/68, 54%) collected data using the mixed methods approach. Only 15% (10/68) of the articles used only qualitative methods. These data collection methods included interviews, focus group discussions, thinking-aloud protocols, cognitive walkthroughs, open-ended discussions, Wizard of Oz, and free-text writing. Approximately two-thirds of the studies (21/68, 31%) used quantitative approaches for data collection during evaluation studies. The System Usability Scale (SUS) outnumbered other usability questionnaires (17/68, 25%) such as the Mobile Application Rating Scale, Net Promoter Score, Single Ease Question, NASA Task Load Index, and Technology Acceptance Model. However, a large percentage (21/68, 31%) of the articles used self-developed surveys or self-reporting questionnaires. Only one-fourth of the articles (16/68, 24%) further implemented statistical analysis including task completion, number of errors, descriptive statistics, or Google Analytics for the assessment of usability. Consequently, only a small proportion (3/68, 4%) of the included studies performed heuristic evaluation as a form of expert evaluation.

## Discussion

### Principal Findings

Our data suggest that the evaluation of wearables for medical purposes was largely conducted without direct integration with mobile apps. Although some studies used smartphones as a means of connecting with the wearables, users were not assigned companion apps for data viewing. This limits the analysis and data visualization capabilities of the data collected within the studies. Wrist-worn devices were the most common type of wearables identified in the studies, indicating the convenience of using this type of wearables for measuring physical activities in a research setting. We also found that most studies (21/68, 47%) reported the use of consumer-grade fitness and activity trackers from Fitbit, and only a handful of the studies (5/68, 7%) implemented medical-grade wearable devices. A small number of the studies (10/68, 15%) investigated the data collection and use of the wearable in the aspects of aftercare of patients, wellness, and rehabilitation.

We noted the absence of standards or guidelines to facilitate the analysis of the usability of wearables, mobile apps, or their combination. Although user- and human-centered design frameworks were mentioned in a few studies (7/68, 10%), they are guiding the design and development of systems and devices focusing on users and their needs but not the usability of these devices. Despite the fact that many wearable technologies were included in the study, no usability evaluations of multiple devices, the combination of devices, or multidevice interfaces were reported.

Only a little more than one-third of the included publications (22/68, 32%) in our review explicitly reported the measurement of usability or perceived usability. Some studies (8/68, 12%) primarily focused on assessing the measurement of *usability* or *perceived usability* as user perceptions of the devices, attitudes, and compliance using different qualitative or quantitative methods [71,89,91,95,97,98,102,129]. However, it is worth noting that these studies encountered a challenge in clearly differentiating the evaluation of the usability of the wearable device from that of the accompanying mobile app. Consequently, the intended purpose of the evaluation may have been limited in these studies. Therefore, we address the term *usability*, a broad term that encompasses various factors including technology and user acceptability. Studies might have reported on usability alone either due to missing expertise or due to a high-level summarization of various aspects they investigated. To help mitigate this, we incorporated and synthesized a set of attributes or subattributes to measure the capability and performance of a system based on the extracted data.

Our analysis showed that a subset of usability studies lacked testing with the intended target group and instead relied on healthy adults as participants. Despite the absence of explicit acknowledgment of this limitation in the included studies, it raises concern regarding the extent to which the devices and apps under investigation adequately address the unique needs and requirements of the target users, particularly individuals with chronic health conditions. This observation is in line with the findings reported in previous studies [135-144], emphasizing the concern regarding the devices and apps under investigation that adequately meet the unique needs and requirements of the target users.

### Comparison With Prior Work

#### Wearables

Most of the studies (45/68, 66%) in our review used consumer-grade wearables. This matches the observations of other studies [145-150] that investigated commercially available wearables and reported a wide variety of purposes, ranging from digital diagnostic tools to sports tracking to remote monitoring. Niknejad et al [151] and Ferreira et al [152] reported that consumer-grade wearables have been used to foster self-awareness among users. In contrast, we observed that the studies using consumer-grade wearables in our corpus focused on their use for monitoring chronic conditions such as diabetes, obesity, cardiology, and cancer.

We found that wearables are gradually being used more widely in health care and clinical settings. As stated in the previous paragraph, this is true for consumer-grade wearables such as Fitbit, Jawbone, Apple, and Garmin [153-157]. However, concerns about the safety, reliability, and accuracy of these devices persist. In their work, Piwek et al [146] raised concerns about wearables in terms of user safety, emphasizing the need to better address the reliability and security of the data collected from these devices. Considering the inherent lack of emphasis on user safety in consumer wearables, it is imperative to acknowledge the importance of adhering to standard safety and privacy protocols. This includes ensuring ethical transparency and providing appropriate education to users regarding the privacy and information security risks they may encounter when using such devices [24,158,159]. In addition, the use of consumer wearables in health care settings remains somewhat ambivalent at present [160,161]. Although our review did not specifically address these concerns, we acknowledge that these factors significantly affect the usability of wearable technologies, whether used independently or with companion mHealth apps. Similar to Piwek et al [146], who pointed out acceptance challenges of wearables concerning safety and security, we believe that a structured framework with clear definitions and well-defined methods would allow bringing wearables into more diverse practices in the health care system, encouraging a broader adoption and implementation of use cases with high and tested usability.

Studies by Niknejad et al [151], Dimou et al [162], and Yang et al [163] proposed different categorization approaches for wearables, considering factors such as industry relevance (eg, health care or fashion) or wearable placement on the body. However, owing to the wide variety of wearables available in the market, establishing a standardized classification or hierarchy for these different types of wearables becomes challenging. Thus, to help designers and developers, a standardized classification or hierarchy would be helpful when selecting wearables for specific use cases.

#### Usability Attributes

Many studies in our corpus did not explicitly state the usability attributes they evaluated. Some mentioned generic terms such as *usability* or *user experience*, but did not define them further for their specific cases. As we have argued, more specific usability attributes can facilitate the development of more appropriate requirements and clearer identification of problems in usability studies. This matches observations in related areas. Meyer et al [164] analyzed usability evaluation practices in wearable robotics and recommended better distinguishing between the different usability dimensions and including qualitative measures for identifying a wider range of usability issues. Chiauzzi et al [139] examined the use of wearable devices for long-term chronic disease management. Patient concerns regarding technical difficulties and the appeal of the devices were identified, but their investigation did not address usability attributes such as device comfort and usefulness. Furthermore, the authors emphasized the importance of wearables being perceived as usable and generating comprehensible data to facilitate wider adoption among patients.

Among the 29 identified attributes in our review, 6 (21%) attributes were found to be more suitable for capturing the quality or product-related aspects of the technologies investigated in this study (Table 5). However, determining the most appropriate attributes for wearables and their associated mobile apps can be challenging because of the potential overlap between the product and inherent attributes, such as effectiveness and satisfaction (Table 5). This finding is consistent with Bakhshian and Lee [165], who argued that consumers’ attitudes and purchase intentions toward wearable technology are influenced by both product attributes and inherent attributes, including functional, expressive, and esthetic characteristics. In contrast, other studies have explored the design attributes and their influence on user interactions and acceptance of different types of wearables for specific use cases, such as electroencephalogram systems [166], autism spectrum disorders [167], sports applications [168], and haptic feedback wearable robots [169]. The importance of assessing usability based on user interactions with wearable devices and their associated app remains crucial, amid the emphasis on the design and quality attributes of wearables.

Our data highlight the importance of considering supplementary attributes such as wearability, perceived usefulness, and connectivity when evaluating individual wearables and companion mobile apps. Consistent with our findings, the existing literature also emphasizes the significance of incorporating auxiliary attributes beyond the conventional usability factors such as effectiveness and satisfaction to enhance the acceptance of wearable devices. The aforementioned supplementary attributes identified in the literature include characteristics such as comfort, user-friendliness, affordability, useful features, and appealing design [33-35,136]. It is imperative to incorporate these attributes into the evaluation process to ensure comprehensive assessments of usability and user acceptance in both wearables and companion mobile apps. Although a few studies provide a general overview of wearable attributes related to design and product quality, there is limited research that specifically focuses on the usability attributes of wearables. Although some reviews have identified specific usability attributes for mHealth apps used in various use cases [41,170-176], these are insufficient when evaluating the combined use of wearables and companion apps because of the complex and multifaceted features and interactions involved.

Our results revealed diverse informal terminology used to describe usability, performance, and quality aspects of the technologies examined in the studies. This variability in terminology posed challenges in accurately distinguishing and classifying the terms based on their usability characteristics. We adopted an existing usability taxonomy from the literature to address this issue and ensure consistency in data interpretation [66].

#### Usability Evaluation Methods and Frameworks

Our analysis revealed that only 9 studies incorporated the user- and human-centered design framework in the design and development of prototypes. These studies specifically targeted specific user populations and assessed usability attributes such as satisfaction, ease of use, and effectiveness as part of their evaluation process. Although usability frameworks are available individually for the design and development of wearables and mobile apps [32,177-182], a usability evaluation framework for the combination of these technologies or multi-interface devices is unavailable.

In our scoping review, most of the included studies (57/68, 84%) used qualitative methods, with interviews being the primary method. This corresponds with other studies reporting on evaluation methodologies [183-185]. A survey on the evaluation of physical activity apps highlighted that a substantial number of studies specifically used a mixed methods approach, including randomized controlled trials, to assess the acceptability and evaluate the usability of wearable technologies, with or without companion mobile apps [186]. This approach has gained popularity because of its comprehensive nature and ability to capture diverse perspectives. Among the combinations of methods used, questionnaires and interviews have emerged as commonly used techniques [75,187-189].

Among the 68 studies included in our review, only one-third (20/68, 31%) used a standardized usability questionnaire to evaluate perceived usability. These questionnaires comprise a predefined set of questions presented in a specific order and format, with established scoring rules based on respondents’ answers [190]. The SUS questionnaire [191] was used most frequently among the studies analyzed. Although SUS was originally designed as a generic tool for usability assessment across a broad range of digital interfaces and software apps, it may not include items tailored to the specific characteristics and challenges posed by wearable technology. Wearables often involve prolonged and continuous interaction with the user, making aspects such as device comfort and user experience crucial; however, these aspects are not comprehensively addressed by the SUS. Researchers have proposed different adaptations of usability questionnaires tailored to assess the usability of wearables or mobile apps [192-194]. Although this helps to better evaluate the particularities of wearables and mobile apps, they lack items assessing novel usability considerations such as ergonomics, comfort, real-time data feedback, and interaction with the wearable independently or combined with their associated mobile app, which has been identified as important factors by studies [33,73,195,196]. The investigation of these aspects, which are crucial for most systems and apps, has been limited and partial. These observations may be attributed to various factors, including inadequate awareness of available usability questionnaires, the perception that these questionnaires do not align with their specific study, or the belief that the items or constructs within the questionnaires do not adequately reflect the purpose of their evaluation.

#### Concluding Analysis

We have shown that health care professionals and the medical technology industry acknowledge the importance of high or adequate usability in new medical equipment, including wearables and mobile apps. In addition, our results highlight the shortcomings in the evaluation and reporting of usability for wearable technologies, necessitating further research on human factors and usability. Literature reviews emphasize concerns regarding the lack of standardized study methodology reporting, guidelines for evaluating usability, and the absence of frameworks or theories for designing comprehensive usability assessments [151,197-199]. According to the reviews conducted by Khakurel et al [27] and Keogh et al [144], the current literature lacks a comprehensive usability evaluation method that effectively addresses usability issues throughout the entire life cycle of a wearable device, from the early development stages to product release. Considering the significance of reliability and wearability in wearable devices, it is imperative to establish traceability in the usability evaluation process. Although researchers are actively engaged in assessing usability, further research is required to identify potential usability attributes, develop suitable evaluation methods and frameworks, and successfully integrate these effective assessments into practice.

### Limitations

The goal of this scoping review was to investigate wearable devices and their frequency of use in studies as well as their combination with mHealth apps within the medical domain. Our findings may not completely capture the breadth of usability attributes and their effectiveness in wearables across different contexts. Wearables and their companion apps have demonstrated utility in various use cases and recreational activities, including industrial settings, gaming, museums, and entertainment. By limiting our search to health care–related keywords, we may have excluded valuable insights and perspectives from these alternative domains. Future research could broaden the search criteria to include diverse contexts and use cases beyond health care. This will allow for a more holistic exploration of the potential and effectiveness of usability attributes across different industries and settings.

This study did not aim to synthesize evidence on the effectiveness of usability evaluation methods. Instead, it focused on capturing the diversity of the available literature, encompassing various objectives, critical usability measures, and methods. Consequently, this study primarily serves as an exploratory investigation and provides suggestions for future research in the field.

### Conclusions

Our scoping review sheds light on the types and categories of wearable devices, frequency of wearables used in the medical context, their use cases, and the evaluation of their usability. With a wide array of wearables and mHealth apps available, health care providers and manufacturers face the challenge of selecting devices and apps that are effective and user-friendly. The evaluation of usability is crucial for ensuring user engagement and the success of these technologies. As our scoping review shows, there is a lack of standardized frameworks for classifying usability attributes and their subattributes as well as structured evaluation guidelines for wearable technologies. This gap in usability and user experience research hinders the understanding of strengths and limitations in the field of wearable technologies. Therefore, further research is needed to address these limitations and enhance the comprehension of researchers in this field.

## Appendix

Multimedia Appendix 1 PRISMA-ScR (Preferred Reporting Items for Systematic Reviews and Meta-Analyses extension for Scoping Reviews) checklist.

Multimedia Appendix 2 Search strategies for the 4 databases.

Multimedia Appendix 3 List of the included studies and extracted information.

Multimedia Appendix 4 Short description of the different attributes in Tables 4 and 5.

## Abbreviations

ISO: International Organization for Standardization
mHealth: mobile health
PICO: Population, Intervention, Comparison, and Outcome
PRISMA-ScR: Preferred Reporting Items for Systematic Reviews and Meta-Analyses extension for Scoping Reviews
RQ: research question
SUS: System Usability Scale
