# Supplementary material for: Attributes, Methods, and Frameworks Used to Evaluate Wearables and Their Companion mHealth Apps: Scoping Review
Source: JMIR Mhealth Uhealth. 2024 Apr 5;12:e52179. doi: 10.2196/52179 (PMC11031706; doi:10.2196/52179)
Supplement: Multimedia Appendix 2 [file mhealth_v12i1e52179_app2.docx]

**PICO Model** (**P**opulation, **I**ntervention, **C**ontrol, and **O**utcomes)

| P | Population of Interest | Patient or the problem to be addressed |
| --- | --- | --- |
| I | Intervention | Exposure to be considered– treatments/ tests |
| C | Control/ Comparison | Control or comparison intervention treatment/placebo/standard of care |
| O | Outcome | Outcome of interest |

| **P** | Wearable device/ technology  Fitness Tracker/ Activity trackers  Physiological data  Heart rate/ Sleep monitoring |
| --- | --- |
| **I** | Mobile devices/ applications  Telemedicine/ mHealth |
| **C** | *Comparison (C) is eliminated since our focus is not on comparative studies or controlled exposure* |
| **O** | User-Computer Interface  Usability/ User Experience  User Centered Design Process  User Evaluation/ Interaction |

**Strategy:**

| 1 | P |  |
| --- | --- | --- |
| 2 | I |  |
| 3 | O |  |
| 4 | 1 AND 2 AND 3 |  |
| 5 | NOT | Virtual Reality, Augmented Reality, Google Glass |
| 6 | (1 AND 2 AND 3) NOT 5 |  |

**Queried databases:**

- PubMed
- Web of Science Core Collection
- ACM Digital Library
- IEEE Xplore

**PubMed**

| **Number of hits** | **Date** |
| --- | --- |
| 146 | December 2020 |
| 51 | December 2021 |

| **P** | **Results** | **not exclusively relevant terms** |
| --- | --- | --- |
| **"Wearable Electronic Devices"[Mesh] OR**  Wearable Technolog*[tw] OR  Wearable Device*[tw] OR |  | Wearable Electronic Device*[tw] OR  Electronic Skin*[tw] OR  electronic textile*[tw] OR  smart textile*[tw] OR |
| **"Fitness Trackers"[Mesh] OR**  Fitness Tracker*[tw] OR  Activity Tracker*[tw] OR |  |  |
|  |  | **"Electromyography"[Mesh] OR**  Electromyograph*[tw] OR  Electromyogram*[tw] OR |
|  |  | Gyroscope*[tw] OR |
|  |  | Accelerometer*[tw] OR |
|  |  | **“Motion”[Mesh] OR**  Motion*[tw] OR |
|  |  | **“track and field”[Mesh] OR**  Track and field*[tw] OR |
| Sleep Monitoring [tw] OR  Somnography*[tw] OR |  | **"Polysomnography"[Mesh] OR** |
| **Physiological data** |  | **"Monitoring, Physiologic"[Mesh] OR**  Patient Monitoring*[tw] OR |
| Heart rate variability*[tw] |  |  |

| **I** | **Results** | **Excluded terms – not exclusively relevant** |
| --- | --- | --- |
| **"Mobile Applications"[Mesh] OR**  Mobile App*[tw] OR  mobile device*[tw] OR  mobile phone*[tw] OR  smartphone*[tw] OR |  | Portable Electronic App*[tw] OR  Portable Software App*[tw] OR  Portable Electronic*[tw] OR  "Portable Software"[tw] OR  mobile technolog*[tw] OR |
|  |  | **"Medical Informatics Applications"[Mesh] OR**  Medical Informatics App*[tw] OR  Medical Informatics Application*[tw] OR |
| **"Telemedicine"[Mesh] OR**  Mobile Health*[tw] OR  mHealth*[tw] OR  m Health*[tw] |  | eHealth*[tw] OR  e Health*[tw] OR  mobile medical app*[tw] |

| **O** | **Results** | **Excluded terms – not exclusively relevant** |
| --- | --- | --- |
| **"User-Computer Interface"[Mesh] OR**  Usab*[tw] OR  "User Interface"[tw] OR  User Experience*[tw] OR  “user centered”[tw] OR  user interface*[tw] OR  user evaluation*[tw] OR  user interaction*[tw] |  | "User Computer Interface"[tw] OR  user oriented*[tw] |

**(("Wearable Electronic Devices"[Mesh] OR Wearable Technolog*[tw] OR Wearable Device*[tw] OR "Fitness Trackers"[Mesh] OR Fitness Tracker*[tw] OR Activity Tracker*[tw] OR Sleep Monitoring*[tw] OR Somnography*[tw] OR Physiological data[tw] OR Heart rate variability*[tw]) AND ("Mobile Applications"[Mesh] OR Mobile App*[tw] OR mobile device*[tw] OR mobile phone*[tw] OR smartphone*[tw] OR "Telemedicine"[Mesh] OR Mobile Health*[tw] OR mHealth*[tw] OR m Health*[tw]) AND ("User-Computer Interface"[Mesh] OR Usab*[tw] OR User Experience*[tw] OR "User Interface"[tw] OR "user centered"[tw] OR user evaluation*[tw] OR user interaction*[tw]))**

**Web of Science**

| **Number of hits** | **Date** |
| --- | --- |
| 56 | December 2020 |
| 8 | December 2021 |

| **P** | **Results** | **Excluded terms – not exclusively relevant** |
| --- | --- | --- |
| (TS=("Wearable Electronic Devices" OR "Wearable Technology" OR "Wearable Device" OR "Fitness Trackers*" OR "Activity Tracker*" OR "Sleep Monitoring" OR "Somnography" OR "Physiological data" OR "Heart rate variability")  ) |  | "Electronic Skin*" OR  Electromyograph*" OR  "Electromyogram*" OR  "Gyroscope*" OR  "Accelerometer*" OR  "electronic textile*" OR  "smart textile*" |

| **I** | **Results** | **Excluded terms – not exclusively relevant** |
| --- | --- | --- |
| (TS= ("Mobile Applications*" OR "Mobile App*" OR "mobile device*" OR "mobile phone*" OR "smartphone*" OR "Telemedicine*" OR "Mobile Health*" OR "mHealth*" OR "m Health*")) |  | "Portable Electronic*" OR  "Portable Software" OR  "Medical Informatics App*" OR  "eHealth*" OR  "e Health*" OR  "mobile medical app*"  "mobile technolog*" OR |

| **O** | **Results** | **Excluded terms – not exclusively relevant** |
| --- | --- | --- |
| (TS= ("User Computer Interface*" OR "user interface*" OR "Usab*" OR "User Experience*" OR (“user centered*”/ NEAR "user centred*") OR "user evaluation*" OR "user interaction*")) |  | "user oriented*" |

**(TS=("Wearable Electronic Devices" OR "Wearable Technology" OR "Wearable Device" OR "Fitness Trackers*" OR "Activity Tracker*" OR "Sleep Monitoring" OR "Somnography" OR "Physiological data" OR "Heart rate variability")) AND (TS=("Mobile Applications" OR "Mobile App" OR "mobile device" OR "mobile phone" OR "smartphone" OR "Telemedicine" OR "Mobile Health" OR "mHealth" OR "m Health")) AND (TS=("User-Computer Interface" OR "Usability" OR "User Experience" OR "User Interface*" OR "user centered" OR "user evaluation" OR "user interaction"))**

**ACM Digital Library**

| **Number of hits** | **Date** |
| --- | --- |
| 8 | December 2020 |
| 10 | December 2021 |

| **P** | **Results** | **Excluded terms – not exclusively relevant** |
| --- | --- | --- |
| [[Abstract: "wearable electronic devices"] OR [Abstract: "wearable technology"] OR [Abstract: "wearable device"] OR [Abstract: "fitness trackers"] OR [Abstract: "activity tracker"] OR [Abstract: "sleep monitoring"] OR [Abstract: "somnography"] OR [Abstract: "physiological data"] OR [Abstract: "heart rate variability"]] |  | "Electronic Skin*" OR  [Abstract: "electronic textile*"] OR [Abstract: "smart textile*"] OR  [Abstract: "electromyography*"] OR [Abstract: "electromyogram*"] OR  [Abstract: "gyroscope*"] OR  [Abstract: "accelerometer*"] |

| **I** | **Results** | **Excluded terms – not exclusively relevant** |
| --- | --- | --- |
| [Abstract: "mobile applications*"] OR [Abstract: "mobile app*"] OR [Abstract: "mobile device*"] OR [Abstract: "mobile phone*"] OR [Abstract: "smartphone*"] OR [Abstract: "telemedicine*"] OR [Abstract: "mobile health*"] OR [Abstract: "mhealth*"] OR [Abstract: "m health*"] |  | "Portable Electronic*" OR  "Portable Software" OR  "Medical Informatics App*" OR  "eHealth*" OR  "e Health*" OR  "mobile medical app*" OR  [Abstract: "mobile technology*"] OR |

| **O** | **Results** | **Excluded terms – not exclusively relevant** |
| --- | --- | --- |
| [Abstract: "user computer interface*"] OR [Abstract: "user interface*"] OR [Abstract: "usability*"] OR [Abstract: "user experience*"] OR [Abstract: "user centered*"] OR [Abstract: "user centred*"] OR [Abstract: "user evaluation*"] OR [Abstract: "user interaction*"] |  | "user oriented*" |

**[[Abstract: "wearable electronic devices"] OR [Abstract: "wearable technology"] OR [Abstract: "wearable device"] OR [Abstract: "fitness trackers"] OR [Abstract: "activity tracker"] OR [Abstract: "sleep monitoring"] OR [Abstract: "somnography"] OR [Abstract: "physiological data"] OR [Abstract: "heart rate variability"]] AND [[Abstract: "mobile applications"] OR [Abstract: "mobile app"] OR [Abstract: "mobile device"] OR [Abstract: "mobile phone"] OR [Abstract: "smartphone"] OR [Abstract: "telemedicine"] OR [Abstract: "mobile health"] OR [Abstract: "mhealth"] OR [Abstract: "m health"]] AND [[Abstract: "user-computer interface"] OR [Abstract: "usability"] OR [Abstract: "user experience"] OR [Abstract: "user interface"] OR [Abstract: "user centered"] OR [Abstract: "user evaluation"] OR [Abstract: "user interaction"]]**

**IEEE Xplore**

| **Number of hits** | **Date** |
| --- | --- |
| 82 | December 2020 |
| 7 | December 2021 |

| **P** | **Results** | **Excluded terms – not exclusively relevant** |
| --- | --- | --- |
| ("Wearable Electronic Devices" OR "Wearable Technology" OR "Wearable Device" OR "Fitness Trackers*" OR "Activity Tracker*" OR "Sleep Monitoring" OR "Somnography" OR "Physiological data" OR "Heart rate variability") |  | "Electronic Skin*" OR  Electromyograph*" OR  "Electromyogram*" OR  "Gyroscope*" OR  "Accelerometer*" OR  "electronic textile*" OR  "smart textile*" |

| **I** | **Results** | **Excluded terms – not exclusively relevant** |
| --- | --- | --- |
| ("Mobile Applications" OR "Mobile App" OR "mobile device" OR "mobile phone" OR "smartphone" OR "Telemedicine" OR "Mobile Health" OR "mHealth" OR "m Health") |  | "Portable Electronic*" OR  "Portable Software" OR  "Medical Informatics App*" OR  "eHealth*" OR  "e Health*" OR  "mobile medical app*" OR  "mobile technology*" OR |

| **O** | **Results** | **Excluded terms – not exclusively relevant** |
| --- | --- | --- |
| ("Usab*" OR "User Computer Interface" OR "User Experience" OR "user centered" OR "user evaluation" OR "user interaction" OR "user interface") |  | "user oriented*" |

**(("Wearable Electronic Devices" OR "Wearable Technology" OR "Wearable Device" OR "Fitness Trackers*" OR "Activity Tracker*" OR "Sleep Monitoring" OR "Somnography" OR "Physiological data" OR "Heart rate variability") AND ("Mobile Applications" OR "Mobile App" OR "mobile device" OR "mobile phone" OR "smartphone" OR "Telemedicine" OR "Mobile Health" OR "mHealth" OR "m Health") AND ("User-Computer Interface" OR "Usability" OR "User Experience" OR "User Interface*" OR "user centered" OR "user evaluation" OR "user interaction"))**
