# Supplementary material for: Attributes, Methods, and Frameworks Used to Evaluate Wearables and Their Companion mHealth Apps: Scoping Review
Source: JMIR Mhealth Uhealth. 2024 Apr 5;12:e52179. doi: 10.2196/52179 (PMC11031706; doi:10.2196/52179)
Supplement: Multimedia Appendix 4 [file mhealth_v12i1e52179_app4.docx]

**Short description of the usability attributes identified in the scoping review and the usability taxonomy**

| **Attributes identified in the scoping review** | **Descriptions** | **References** |
| --- | --- | --- |
| Accuracy | The precision and comprehensiveness with which users attain predefined objectives and the capability to furnish accurate outcomes or impacts. | ISO 9241-11 (1998) Ergonomic requirements for office work with visual display terminals (VDTs) Part 11: Guidance on Usability. ISO. |
|  |  | International Organization for Standardization/International Electrotechnical Commission, 2001. ISO/IEC9126-1 Standard, Software Engineering, Product Quality, Part 1: Quality Model, Geneva: Author. |
| Aesthetics | Refers to the system's ability to please the user in sensory terms, encompassing visual, acoustic, tactile, olfactory, and gustatory aesthetics. It quantifies the extent to which a user interface facilitates enjoyable and satisfying interaction for the user. The concept of aesthetics also connects to beauty in nature and art, signifying something that rejuvenates both body and mind, awakening the senses. | Alonso-Ríos, D., Vázquez-García, A., Mosqueira-Rey, E. and Moret-Bonillo, V., 2009. Usability: a critical analysis and a taxonomy. International journal of human-computer interaction, 26(1), pp.53-74. |
|  |  | https://iso25000.com/index.php/en/iso-25000-standards/iso-25010/61-usability |
|  |  | O. Conolly and B. Haydar, "Aesthetic Principles," The British Journal of Aesthetics, vol. 43, pp. 114125, 2003. |
| Attitude | Refers to the user/individual's assessment regarding the appeal and desirability of engaging with a specific information system or mobile application. | Davis, F. D. “Perceived Usefulness, Perceived Ease of Use, and User Acceptance of Information Technology,” MIS Quarteri’y (13:3), September 1989, pp.3 19-339. |
| Attractiveness | The software product's ability to visually engage users, manifested through factors such as color utilization and graphical design. | ISO 9126-1, 2000. Software Engineering––Product Quality––Part 1: Quality Model |
| Clarity | The ease with which the system is comprehensible to the mind and senses, encompassing the clarity of elements, including sensorial and semantic aspects, the clarity of structure, involving formal and conceptual elements, and clarity in functioning, pertaining to user and system task execution. | Mosqueira-Rey, E., Alonso-Ríos, D. and Moret-Bonillo, V., 2009, October. Usability taxonomy and context-of-use taxonomy for usability analysis. In *2009 IEEE International Conference on Systems, Man and Cybernetics* (pp. 812-817). IEEE. |
|  |  | Alonso-Ríos, D., Vázquez-García, A., Mosqueira-Rey, E. and Moret-Bonillo, V., 2009. Usability: a critical analysis and a taxonomy. *International journal of human-computer interaction*, *26*(1), pp.53-74. |
| Cognitive Load | Denotes the mental effort required for understanding, quantifying the demand on working memory as "brain power" or the utilization of memory slots. It represents the total mental activity on working memory at a specific instance, indicating the load imposed by a user interface in terms of the mental resources needed to operate the system. | https://www.usability.gov/what-and-why/glossary/c/index.html |
|  |  | https://www.nngroup.com/articles/minimize-cognitive-load/ |
|  |  | Cooper, Graham. “Research into Cognitive Load Theory and Instructional Design at UNSW.” University of New South Wales. 9 Aug 2004 |
| Controllability | Revolves around the system's adaptability to user preferences and task control and relates to user-driven system use. Under the umbrella of controllability, configurability allows personalization of technical and formal aspects, while overall workflow controllability pertains to user-directed task execution, including options for task approaches and action reversibility. | Mosqueira-Rey, E., Alonso-Ríos, D. and Moret-Bonillo, V., 2009, October. Usability taxonomy and context-of-use taxonomy for usability analysis. In *2009 IEEE International Conference on Systems, Man and Cybernetics* (pp. 812-817). IEEE. |
|  |  | Alonso-Ríos, D., Vázquez-García, A., Mosqueira-Rey, E. and Moret-Bonillo, V., 2009. Usability: a critical analysis and a taxonomy. *International journal of human-computer interaction*, *26*(1), pp.53-74. |
| Data Quality | Assessed by its ability to accurately predict system usability in practical scenarios, its efficiency in analysis, and the effectiveness of responses. Defining data quality involves evaluating intrinsic data characteristics and considering extrinsic factors like usability and usefulness for various data consumers. | Danielson, David R. "Usability barriers." In *Encyclopedia of Human Computer Interaction*, pp. 652-660. IGI Global, 2006. |
|  |  | Shanks, Graeme, and Brian Corbitt. "Understanding data quality: Social and cultural aspects." In *Proceedings of the 10th Australasian conference on information systems*, vol. 785. Victoria University of Wellington, New Zealand, 1999. |
| Ease of Use/ Perceived ease of use (effort expectancy, easiness, self-descriptiveness, self-efficacy) | The fundamental concept, signifying the simplicity with which users can operate a product and Perceived Ease of Use is defined as the extent to which an individual believes that using a specific system would involve minimal effort. | https://www.interaction-design.org/literature/topics/ease-of-use |
|  |  | F.D. Davis, Perceived Usefulness, Perceived Ease of Use, and User Acceptance of Information Technology, MIS Quarterly, 13, No. 3, (1989). |
| Effectiveness (user errors, ease of executing a task, task completion, task completeness | The degree to which users accomplish their specified goals with accuracy and completeness, assessing the success and correctness of the tasks undertaken. | Zhang, D. and Adipat, B., 2005. Challenges, methodologies, and issues in the usability testing of mobile applications. International journal of human-computer interaction, 18(3), pp.293-308. |
|  |  | https://econsultancy.com/the-attributes-of-usability-and-how-to-exploit-them/ |
|  |  | Quesenbery, W. (2001). What does usability mean: Looking beyond ‘ease of use’. Proceedings of the 18th Annual Conference Society for Technical Communications. |
|  |  | https://www.iso.org/obp/ui/#iso:std:iso:9241:-11:ed-2:v1:en |
|  |  | https://www.usability.gov/what-and-why/glossary/e/index.html |
| Efficiency (task time) | The system's ability to yield appropriate results in proportion to invested resources. It involves the speed and accuracy with which users can achieve their tasks once they have learned the system, measuring the relationship between resources used and results achieved, considering metrics such as the number of clicks, keystrokes, or total time spent on a task. | Alonso-Ríos, D., Vázquez-García, A., Mosqueira-Rey, E. and Moret-Bonillo, V., 2009. Usability: a critical analysis and a taxonomy. International journal of human-computer interaction, 26(1), pp.53-74 |
|  |  | Nielsen,J.: Usability engineering. Academic Press(1993) |
|  |  | https://www.iso.org/obp/ui/#iso:std:iso:9241:-11:ed-2:v1:en |
|  |  | Zhang, D. and Adipat, B., 2005. Challenges, methodologies, and issues in the usability testing of mobile applications. International journal of human-computer interaction, 18(3), pp.293-308. |
|  |  | Quesenbery, W. (2001). What does usability mean: Looking beyond ‘ease of use’. Proceedings of the 18th Annual Conference Society for Technical Communications. |
| Engagement | Entails crafting a visually appealing, inviting, and trustworthy design that motivates users, fostering active participation and ensuring a positive experience. It encompasses attributes such as challenge, positive affect, endurability, aesthetic appeal, attention, feedback, variety/novelty, interactivity, and perceived user control. An engaging interface is marked by its pleasant, satisfying, and interesting qualities, shaped by visual design, multimedia elements, and subtle aspects that capture and sustain the user's attention and interest. | https://www.usability.de/en/usability-user-experience/glossary/user-engagement.html |
|  |  | O'Brien, H.L. and Toms, E.G. (2008), What is user engagement? A conceptual framework for defining user engagement with technology. J. Am. Soc. Inf. Sci., 59: 938-955. https://doi.org/10.1002/asi.20801 |
|  |  | Quesenbery, W. (2004). Balancing the 5Es: Usability. Cutter IT Journal, 17 (2), 4–11. |
|  |  | Quesenbery, W. (2001). What does usability mean: Looking beyond ‘ease of use’. Proceedings of the 18th Annual Conference Society for Technical Communications. |
|  |  | https://www.usability.gov/what-and-why/glossary/e/index.html |
| Error tolerance | Refers to the system's capacity to maintain a low error rate, ensuring users commit few errors during system use, and facilitating easy recovery from any errors made. This attribute aims to prevent catastrophic errors and encompasses any actions that deviate from the intended goal. Measurement involves counting user actions leading to unintended outcomes during specified tasks, considering severity levels and ease of correction. Use error robustness, a revised interaction principle, emphasizes a system's ability to shield users from errors. | Chen, Yu-Hui, Carol Anne Germain, and Abebe Rorissa. "Defining usability: How library practice differs from published research." *portal: Libraries and the Academy* 11, no. 2 (2011): 599-628. |
|  |  | Nielsen,J.: Usability engineering. Academic Press(1993) |
|  |  | https://www.iso.org/obp/ui/#iso:std:iso:9241:-11:ed-2:v1:en |
|  |  | Zhang, D. and Adipat, B., 2005. Challenges, methodologies, and issues in the usability testing of mobile applications. International journal of human-computer interaction, 18(3), pp.293-308. |
|  |  | https://www.usability.de/en/usability-user-experience/glossary/error-tolerance.html |
|  |  | https://iso25000.com/index.php/en/iso-25000-standards/iso-25010/61-usability |
| Functionality | Refers to the fundamental purpose of a product or service, encompassing a set of attributes related to the existence of functions and their specified properties. These functions aim to fulfill stated or implied needs and include aspects such as suitability, accuracy, interoperability, security, and functionality compliance. | https://www.iso.org/standard/35733.html |
| Hedonic motivation | The inclination to undertake actions that enhance positive experiences or diminish negative experiences. Rooted in the general principle of human behavior and well-being, it reflects the tendency to initiate actions leading to the pursuit of pleasure and avoidance of pain. In the realm of technology, hedonic motivation gauges the degree of an individual's perceived enjoyment, irrespective of performance consequences, while hedonic quality encompasses dimensions like originality and beauty, detached from task-related goals. | Kaczmarek, L.D. (2017). Hedonic Motivation. In: Zeigler-Hill, V., Shackelford, T. (eds) Encyclopedia of Personality and Individual Differences. Springer, Cham. https://doi.org/10.1007/978-3-319-28099-8_524-1 |
|  |  | Gray, J. A. (1981). A critique of Eysenck’s theory of personality. In H. J. Eysenck (Ed.), A model for personality (pp. 246–276). Berlin: Springer. |
|  |  | Huta, V., & Waterman, A. S. (2014). Eudaimonia and its distinction from hedonia: Developing a classification and terminology for understanding conceptual and operational definitions. Journal of Happiness Studies, 15, 1425–1456. |
|  |  | Iskandar, Yulita Hanum P., and Phoebe Yueng Hee Sia. "Mobile travel apps and generation y in malaysia: An empirical evidence to understanding the factors influencing the intention to use." In Impact of mobile services on business development and e-commerce, pp. 186-210. IGI Global, 2020. |
|  |  | Hassenzahl, Marc. "The effect of perceived hedonic quality on product appealingness." International Journal of Human-Computer Interaction 13, no. 4 (2001): 481-499. |
|  |  | Hassenzahl, Mare, Axel Platz, Michael Burmester, and Katrin Lehner. "Hedonic and ergonomic quality aspects determine a software's appeal." In Proceedings of the SIGCHI conference on Human factors in computing systems, pp. 201-208. 2000. |
|  |  | Oliver M.B., Raney A.A. Entertainment as pleasurable and meaningful: Identifying hedonic and eudaimonic motivations for entertainment consumption. J. Commun. 2011;61:984–1004. doi: 10.1111/j.1460-2466.2011.01585.x. |
| Learnability | Assesses how easily users can perform tasks when initially encountering an interface and how quickly they become proficient through repetition. It is fundamental for users to swiftly learn and use a system, emphasizing their ability to accomplish tasks efficiently and improve performance over time. This trait includes the system's ease of use, discoverability, and the recognition of learned functions. In essence, learnability gauges the user's effectiveness, efficiency, satisfaction, and risk-free learning experience within a specified context of use. | https://www.nngroup.com/articles/measure-learnability/ |
|  |  | https://econsultancy.com/the-attributes-of-usability-and-how-to-exploit-them/ |
|  |  | Nielsen,J.: Usability engineering. Academic Press(1993) |
|  |  | Zhang, D. and Adipat, B., 2005. Challenges, methodologies, and issues in the usability testing of mobile applications. *International journal of human-computer interaction*, *18*(3), pp.293-308. |
|  |  | https://www.usability.de/en/usability-user-experience/glossary/learnability.html |
|  |  | <https://iso25000.com/index.php/en/iso-25000-standards/iso-25010/61-usability> |
|  |  | https://www.usability.gov/what-and-why/glossary/l/index.html |
| Likes and dislike | Pertains to users' satisfaction with achieving pragmatic goals and acceptable perceived outcomes and consequences of use. This trait is shaped by users' perceptions, feelings, and opinions of the product, reflecting cognitive, emotional, and physical dimensions of satisfaction. | http://miageprojet2.unice.fr/@api/deki/files/2222/=ISO_25010.pdf |
|  |  | Rubin, J., 1994. Handbook of Usability Testing, New York: John Wiley. |
|  |  | Bevan,N. Classifying and selecting ux and usability measures. In International Workshop on Meaningful Measures: Valid Useful User Experience Measurement (2008),13–18. |
| Perceived Usefulness (usefulness, utility, performance expectancy, system usefulness) | The extent to which an individual believes that employing a specific system would contribute to enhancing their job performance. A system high in perceived usefulness is one where the user perceives a positive relationship between system use and performance. | F.D. Davis, Perceived Usefulness, Perceived Ease of Use, and User Acceptance of Information Technology, MIS Quarterly, 13, No. 3, (1989). |
| Satisfaction (subjective app quality, survey/ ratings, positive/ negative feedback, opinions/ reactions, participants' experience) | The measure of the extent to which a system, product, or service fulfills the user's needs and expectations, eliciting positive physical, cognitive, and emotional responses. It encompasses subjective satisfaction, representing the user's attitude and feelings of pleasure and interest during the use of the system or application. | Nielsen,J.: Usability engineering. Academic Press(1993) |
|  |  | https://www.iso.org/obp/ui/#iso:std:iso:9241:-11:ed-2:v1:en |
|  |  | Alonso-Ríos, D., Vázquez-García, A., Mosqueira-Rey, E. and Moret-Bonillo, V., 2009. Usability: a critical analysis and a taxonomy. International journal of human-computer interaction, 26(1), pp.53-74. |
|  |  | Zhang, D. and Adipat, B., 2005. Challenges, methodologies, and issues in the usability testing of mobile applications. International journal of human-computer interaction, 18(3), pp.293-308. |
| Technical difficulties | Unforeseen equipment problems such as hardware failures or software bugs that make it difficult or impossible to perform a desired action. | https://en.wikipedia.org/wiki/Technical_Difficulties |
| Trust | The measure of faithfulness a software product provides to its users. User trust is the belief that a system will appropriately support their tasks or goals in a given situation, influencing their adoption, interaction, and compliance. It involves an attitude of confident expectation in situations of risk, where vulnerabilities are not exploited, reflecting a willingness to be vulnerable based on the expectation of the other party's dependable actions. | http://miageprojet2.unice.fr/@api/deki/files/2222/=ISO_25010.pdf |
|  |  | Ahuja, V. 2000. Building trust in electronic commerce, IT Professional 2: 61–63. |
|  |  | Atif, Y. 2002. Building trust in e-commerce, IEEE Internet Computing 6: 18–24 |
|  |  | Cheskin Research and Studio Archetype/Sapient 1999. e-Commerce trust study. Retrieved June 30, 2005 fromhttp://www.cheskin.com/docs/sites/1/report-eComm%20Trust1999.pdf. |
|  |  | Friedman, B., Kahn, P.H., Jr., and Howe, D.C. 2000. Trust online, ACM Communications, 43: 34–40. |
|  |  | Tilson, R., Dong, J., Martin, S., and Kieke, E., 1998. Factors and principles affecting the usability of foure-commerce sites, Proceedings of the 4th Conference on Human Factors & the Web, Basking Ridge,New Jersey. |
|  |  | https://testscience.org/measuring-user-trust/ |
|  |  | Corritore, C. L., Kracher, B., & Wiedenbeck, S. (2003). On-line trust: concepts, evolving themes, a model. International Journal of Human-Computer Studies, 58, (6), 737–758. http://dx.doi.org/10.1016/S1071-5819(03)00041-7 |
|  |  | Mayer, R.C., Davis, J.H. and Schoorman, F.D. (1995), “An integrative model of organizational trust”, Academy of Management Review, Vol. 20 No. 3, pp. 709‐34 |
| User Control | Users often make mistakes or change their minds. Allows them to exit a flow or undo their last action and go back to the system’s previous state. | https://www.nngroup.com/articles/user-control-and-freedom/s |
| User Experience (experience, overall subjective quality, user-friendliness) | Encompasses the overall perception derived from interacting with a product or service, extending beyond mere usage to include aspects like purchase, support, and packaging. It consists of three key components: the product's utility, its usability in achieving user goals, and the enjoyment derived from using it. A high-quality user experience results when these components are comprehensively considered and effectively coordinated. It represents the user's perceptions and responses arising from the actual or anticipated use of a system, product, or service and encompasses various disciplines studying the impact of design on usability and satisfaction. | https://www.usability.de/en/usability-user-experience/glossary/user-experience.html |
|  |  | https://www.iso.org/obp/ui/#iso:std:iso:9241:-11:ed-2:v1:en |
|  |  | https://www.usability.gov/what-and-why/glossary/u/index.html |

**Short description of the Quality and Product attributes identified in the scoping review and the usability taxonomy**

| **Attributes identified in the scoping review** | **Descriptions** | **References** |
| --- | --- | --- |
| Comfort | Refers to the extent of user satisfaction with the physical aspects of interaction with a system, product, or service. | http://miageprojet2.unice.fr/@api/deki/files/2222/=ISO_25010.pdf |
|  |  | https://www.iso.org/standard/35733.html |
| Effectiveness* | The accuracy and completeness with which  the user achieves specific goals. | https://www.iso.org/standard/35733.html |
| Efficiency* | The effort in relation to effectiveness. | https://www.iso.org/standard/35733.html |
| Facilitating conditions | The extent to which an individual perceives the presence of both organizational and technical infrastructure supporting the utilization of a system, coupled with their possession of the necessary knowledge and resources to effectively employ the system. | https://www.igi-global.com/dictionary/facilitating-conditions/10825 |
|  |  | Chan, F. K. Y., Thong, J. Y. L., Venkatesh, V., Brown, S. A., Hu, P.J. H., & Tam, K. Y. (2010). Modeling citizen satisfaction withmandatory adoption of an E-Government technology. Journalof the Association for Information Systems, 11(10), 519- 549.  https://aisel.aisnet.org/jais/vol11/iss10/2. |
| Information quality | Encompasses accuracy, timeliness, relevance, granularity, and general believability of the information. | Tate, M.E., J. Hope, B. Barnes, S. , Perceived Service Quality in aUniversity Web Portal: Revising the E-Qual Instrument. 2007 |
| Reliability | The extent to which a software product can consistently sustain a predetermined level of performance under defined conditions. It encompasses attributes such as maturity, reflecting the software's resistance to failure; fault tolerance, indicating its ability to maintain performance despite faults; and recoverability, pertaining to the system's capacity to restore performance and recover affected data in the event of a failure. | http://miageprojet2.unice.fr/@api/deki/files/2222/=ISO_25010.pdf |
|  |  | ISO/IEC 9126 - 1991 |
| Trust* | The degree to which a user or other stakeholder has confidence that a product or system will behave as intended. | https://www.iso.org/standard/35733.html |
| Satisfaction* | The degree to which user needs are satisfied when a product or system is used in a specified context of use. | https://www.iso.org/standard/35733.html |

* Overlapping attributes also identified as usability attributes
